# Classification and morphology of middle mesial canals of mandibular first molars in a southern Chinese subpopulation: a cone-beam computed tomographic study

**DOI:** 10.1186/s12903-020-01339-5

**Published:** 2020-12-10

**Authors:** Yeqing Yang, Buling Wu, Junkai Zeng, Ming Chen

**Affiliations:** 1 Stomatological Hospital, Southern Medical University, Guangzhou, 510280 Guangdong China; 2 Shenzhen Stomatology Hospital (Pingshan), Southern Medical University, Shenzhen, China; 3 Nanfang Hospital, Southern Medical University, Guangzhou, China; 4 School of Stomatology, Southern Medical University, Guangzhou, China

**Keywords:** Cone-beam computed tomography, Middle mesial canal, Morphology, Curvature

## Abstract

**Background:**

Cone-beam computed tomography (CBCT) was employed to study the morphology and curvature of middle mesial canals (MMCs) of mandibular first molars (MFMs).

**Methods:**

CBCT scanning was performed on MFMs of 1100 patients. Patients' images that met the inclusion criteria were divided into group A (< 40 years old) and group B (≥ 40 years old) for further study. The images were used to study the incidence of MMCs at different ages, measure the curvature of MMCs in the mesiodistal and buccolingual directions using the Schneider method, and observe the anatomical morphology of the mesial root canal system. All statistical analyses were performed by using SPSS 21.0 software. Quantitative data were presented as mean ± standard deviation. Student’s *t*-test was used to calculate the statistical significance. *P* < 0.05 was considered statistically significant.

**Results:**

In 875 patients, 1750 MFM images met the inclusion criteria. Among these cases, 158 MFMs contained an MMC, yielding an incidence rate of 9.03%. The incidence rate of MMCs was 11.22% in group A and 6.61% in group B, and this difference was statistically significant (*P* < 0.05). The curvature in the mesiodistal direction was 29.39 ± 8.53° in group A and 26.06 ± 8.50° in group B, and this difference was also significant (*P* < 0.05). The curved regions in groups A and B were often located in the middle 1/3 of canal. No significant difference in the distance between MMC orifices and mesiobuccal canal orifices or mesiolingual canal orifices was noted (*P* > 0.05). The most common mesial root canal morphological type was type II (3-2) (53.80%).

**Conclusion:**

The incidence of MMCs in MFMs declined as age increased. The canal systems of MMCs were varied and complex, mainly exhibiting an obvious mesiodistal curve. CBCT is an outstanding method to help guide root canal therapy.

*Yeqing Yang and Buling Wu have contribute equally to this article.

## Background

The Mandibular first molars (MFMs) are the first permanent molars to grow into the mouth, and often needed root canal treatment due to pulpitis and periapical periodontitis [1]. The complexity of the root canal system often leads to treatment failure. The success of root canal treatment depends on many factors, such as insufficient understanding the internal anatomy of the root canal and failure to thoroughly clean, shape, and properly seal the root canal [2–4]. The root canal system of the MFM is one of the most complicated in human teeth. For example, the system is characterized by a c-shaped root canal [5], distolingual roots (DLRs) [6], middle mesial canals and the occurrence of fusion root [7]. In 1974, De Pablo OV discovered the existence of a third canal between the mesiobuccal canal and mesiolingual canal in the mesial root of MFM [8]. Pomeranz et al. then divided the canal into three types: independent, confluent, and fin [9]. Later, this type of root canal was also called the “middle mesial canal” [9] and “accessory mesial canal” [10]. Scholars at home and abroad have conducted a large number of studies on MMCs with different methods. However, most of these studies are case reports, and the incidence rate of MMCs ranges from 0.26 to 46.15% [11–16]. The traditional methods of root canal research are mostly performed on teeth in vitro, but the sample size is small and inconvenient. The application of microCT is the most sophisticated method for root canal research, but it is not suitable for clinical detection.

CBCT is a clinical auxiliary imaging unit that has developed rapidly in recent years. Compared with the commonly used apical film and panoramic radiograph, it has the advantages of small radiation dose, high image resolution, three-dimensional reconstruction and simple operation. CBCT images can be analysed by computer and displayed in the sagittal, coronal and axial planes simultaneously, which is especially suitable for oral clinical needs [17, 18]. According to imaging studies on MMC morphology, CBCT can clearly, stereoscopically and intuitively display MMC morphology in coronal, sagittal, axial and 3D images, providing first-hand and reliable clinical imaging data [19–21].

At present, CBCT has been gradually applied to the field of dental pulp diseases as a clearer and more intuitive auxiliary examination method based on X-ray imaging examination. Although many authors have applied CBCT to study MMC, few studies have reported on the morphological classification and curvature of MMC in a southern Chinese subpopulation. The purpose of this paper is to study the curvature and curved regions of MMC in a southern Chinese subpopulation and to explore the correlation with age. Based on previous research and using the mesial root canal types of MFM as a reference, we seek to classify the type of mesial root of MFM in our sample to provide a reference for clinical root canal therapy.

## Methods

### Patients

All patients required radiographic examination of CBCT as part of their dental treatment. The images were taken as part of the routine examination, diagnosis, and treatment planning of patients that included those suffering facial trauma or maxillary sinusitis, who required oral surgery, orthodontic treatment or who needed implant treatment. With the informed consent of the patients, this study was approved by the Medical Ethics Committee of Nanfang Hospital (NFEC-2020-106). CBCT images of 1100 patients in southern China between January 2018 and January 2019 were collected using the CBCT imaging system from the database of the department of oral radiology, Nanfang hospital, Guangzhou. All images were included in the study and further analysis according to the following inclusion criteria:

1. MFMs without periapical disease;
2. MFMs had not been endodontically treated;
3. MFMs have no root canals with open apices or absorption;
4. MFMs exhibit absence of coronal or post and core restorations, which may obscure the imaging study;
5. Good quality CBCT images that are clear and lack artefacts.

### CBCT scanning condition

CBCT images were obtained using a Planmeca Romexis 3D CBCT scanner (Planmeca, Finland). Board-certified radiologists operated the X-ray tube at an accelerated potential with a field-of-view size (FOV) of 8 × 8 cm, a peak voltage of 84 kV, a beam current of 14 mA and an exposure time of 12 s for a full arch. The voxel size was 200 μm × 200 μm, and the minimum layer thickness was 0.15 mm. The detector resolution was 1024 × 1024 pixels, and the pixel size was 127 μm × 127 μm. The image data were exported in DICOM format.

### Analytical method and content

All CBCT images are reconstructed and measured using the image reconstruction software of Planmeca Romexis CBCT. The software is run on a 32-bit Windows 7 system, and a display screen from the Lenovo Company is used. The screen resolution is 1280 × 1024. The whole CT image is observed and analysed in a dark room. Serial axial, coronal, and sagittal CBCT images were thoroughly examined from the pulp orifice to the apex. All of the images were assessed separately by two endodontists. To confirm the reliability of the data, intraexaminer calibration was performed before the experiment. In cases of disagreement, these two endodontists discussed the data until a consensus was reached. An oral radiologist provided guidance when necessary.

CBCT was used to scan the mandibular region of the patient to obtain a continuous image of the mandibular first molar and its periodontal tissue. The obtained image was input into the 3D reconstruction software Materialise Mimics 21.0 in DICOM format, and the appropriate parameters were adjusted to obtain a clear contour. Data were exported to an STL file. Then, 3D reconstruction was performed to build the part of the root canal and obtain the final 3D reconstruction diagram of the root canal.

### MMC classification standard

In CBCT images, the mesial root canal system of MFMs was classified based on classic Vertucci classification and its additional root canal classification [22–25].

Type I (3-3): Pulp chamber bottom have three root canal orifices, and always have three independent root canals, and finally there are three different apical foramens.

Type II (3-2): The pulp chamber bottom has three root canal orifices. Then, these orifices merge into two canals at a certain position of the root canal to yield two apical foramens.

Type III (2-3-1): There are two root canal orifices at the bottom of the pulp chamber. They branch into three independent root canals and finally merge into one root canal that emerges from the same apical foramen.

Type IV (2-3-2): The bottom of the pulp chamber has two root canal orifices that branch into three separate root canals. These canals finally merge into two root canals.

Type V (2-3-2-1): Two root canals are located at the beginning of the pulp chamber and subsequently branch into three independent root canals. Finally, the same apical foramen is formed.

Type VI (1-2-3-2): A root canal opening is located at the bottom of the pulp chamber that subsequently branches into two independent root canals at the upper part of the root canal. Then, the canals divide into three independent root canals and ultimate merge into two root canals.

Type VII (1-3-4-1): Only one root canal orifice is located at the bottom of the pulp chamber. The orifice branches into three independent root canals, subsequently divides into four independent root canals, and finally merges into the same root canal at the apex part of the root canal.

Type VIII (3-2-1): Three different root canal orifices are located at the bottom of the pulp chamber. These orifices merge into two root canals and finally emerge from the same apical foramen.

Type IX (3-2-3-2): At the bottom of the pulp chamber, there are three different root canal orifices that fuse into two root canals, branch into three independent root canals, and finally emerge through two different apical holes out of the root canal system.

Type X (3-4-3-2-1): There are three root canal orifices at the bottom of the pulp chamber. These orifices are divided into four root canals and subsequently merged into three independent root canals. At the apical 1/3 of the root canal, they are merged into two canals, and an apical foramen is finally formed.

If the types of root canal systems found in our experimental sample cannot be identified in the Vertucci classification or additional root canal classifications studied by scholars worldwide, it will be listed separately (Figs. 2, 3, 4).

### Analysis of the curvature and position of the MMC

**Fig. 1:**
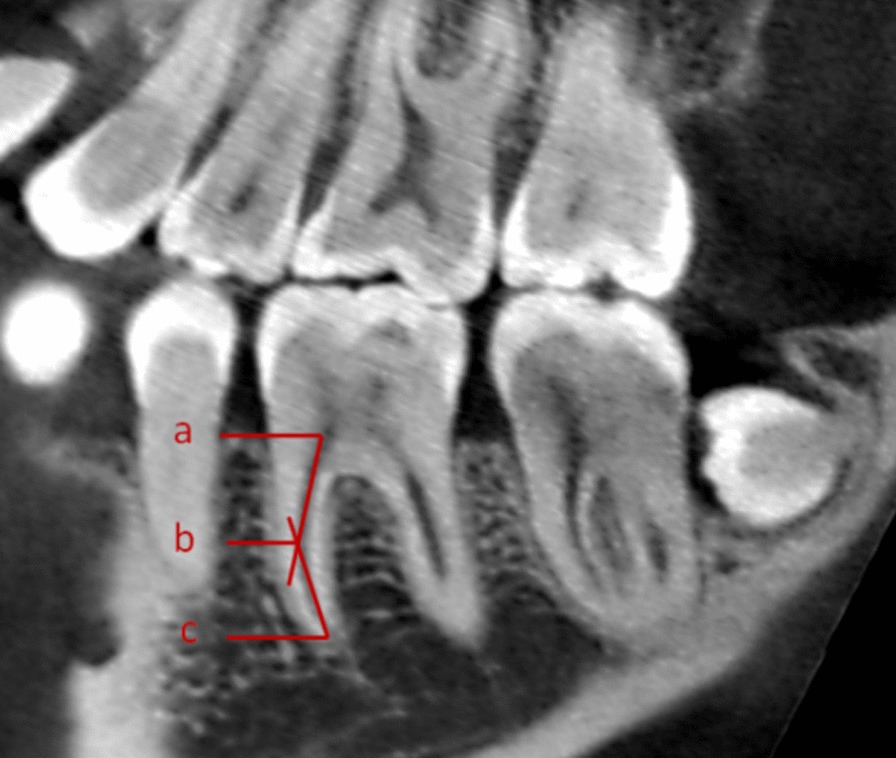
The measurement method for the curvature of root canal. **a** Root canal orifice; **b** ab and bc linear inflection point; **c** apical foramen

In this study, a modified Schneider method was used to measure the curvature of the MMC [26]. This method uses the 3D reconstruction software of the Planmeca Romexis CBCT machine to set the root canal orifice as point a and the apical foramen as point C. A straight line was drawn along the root canal image from point a, and the inflection point was set as point b. The acute angles of the ab and bc lines indicate the root canal curvature. The curvature of the root canal can be classified into three grades: slight curvature was less than or equal to 10°; medium curvature was between 10° and 30°; severe curvature was greater than or equal to 30°. Measurement of root canal curved position was based on the ratio of ab to bc. Set P = ab/bc (Fig. 1). According to the results of P, the curved regions of root canal can be divided into three categories: *P* < 0.5 was class I, and the curved region of the root canal is the upper 1/3; 0.5 < *P* ≤ 2 is class II, and the curved region of the root canal was the middle 1/3; *P* > 2 was class III, and the curved region of root canal was the apical 1/3.

### Statistical analysis

Statistical analysis was performed using SPSS (Version 21.0; SPSS, Inc., Chicago, IL), a statistical software package for Windows. The measurement data were expressed as the mean standard deviation or percentages as appropriate for each measurement calculated at the individual and tooth levels. The chi-square test was used to examine differences among categorical variables, such as age (age < 40 vs. age ≥ 40 years), sex (male vs. female), and side (left vs. right). The mean is compared using t tests with significance set at *P* < 0.05.

## Results

The CBCT images of the MFMs of 1100 patients were observed, and 1750 CBCT images of 875 patients met the inclusion criteria, among which 158 CBCT images contained MMC. The incidence of MMCs in our experimental sample was 9.03%. Among them, there were 81 (51.27%) males and 77 (48.73%) females, and the difference was not statistically significant (*P* > 0.05). The average age of patients with MMCs was (37.9 ± 1.76) years old for males and (33.42 ± 1.52) years old for females (*P* > 0.05). Moreover, there was no significant difference in the distribution of MMCs between the right and left MFMs in both males and females (Table 1).

**Table 1:** The incidence of middle mesial canals in mandibular first molars of different sex

Sex	Single side		Both side	Total
	Left	Right		
Male	39 (24.68%)	37 (23.42%)	5 (3.16%)	81 (51.27%)
Female	36 (22.78%)	37 (23.42%)	4 (2.53%)	77 (48.73%)
Total	149 (94.30%)		9 (5.70%)	158 (9.03%)

In total, 875 patients were divided into group A and group B using the age of 40 years old as the boundary. Group A and B were further subdivided into groups with 10-year intervals. The minimum age was 18 years, and the maximum age was 70 years. In the 18–29 age group of group A, 63 of the 476 teeth contained MMCs, and this root canal incidence was 13.24%. Table 2 demonstrates that as age increases, the incidence of MMCs in MFMs decreased. The difference in the incidence of MMCs between group A and group B was statistically significant (*P* < 0.05) (Table 3).

**Table 2:** Number and percentage of the middle mesial canal in mesial roots of the mandibular first molars by age

	< 40			≥ 40	
	18–29	30–39	40–49	50–59	60–80
Number of specimens/total	63/476	40/442	30/352	17/286	8/194
Incidence	13.24%	9.05%	8.52%	5.94%	4.12%

**Table 3:** Comparison of MMC’s incidence of the MFMs between the group A and group B

Group	Total number of teeth	Number of teeth with MMC	Incidence (%)
Group A	918	103	11.22*
Group B	832	55	6.61
Total	1750	158	9.03

*Compared with group B, *P* < 0.05

**Fig. 2:**
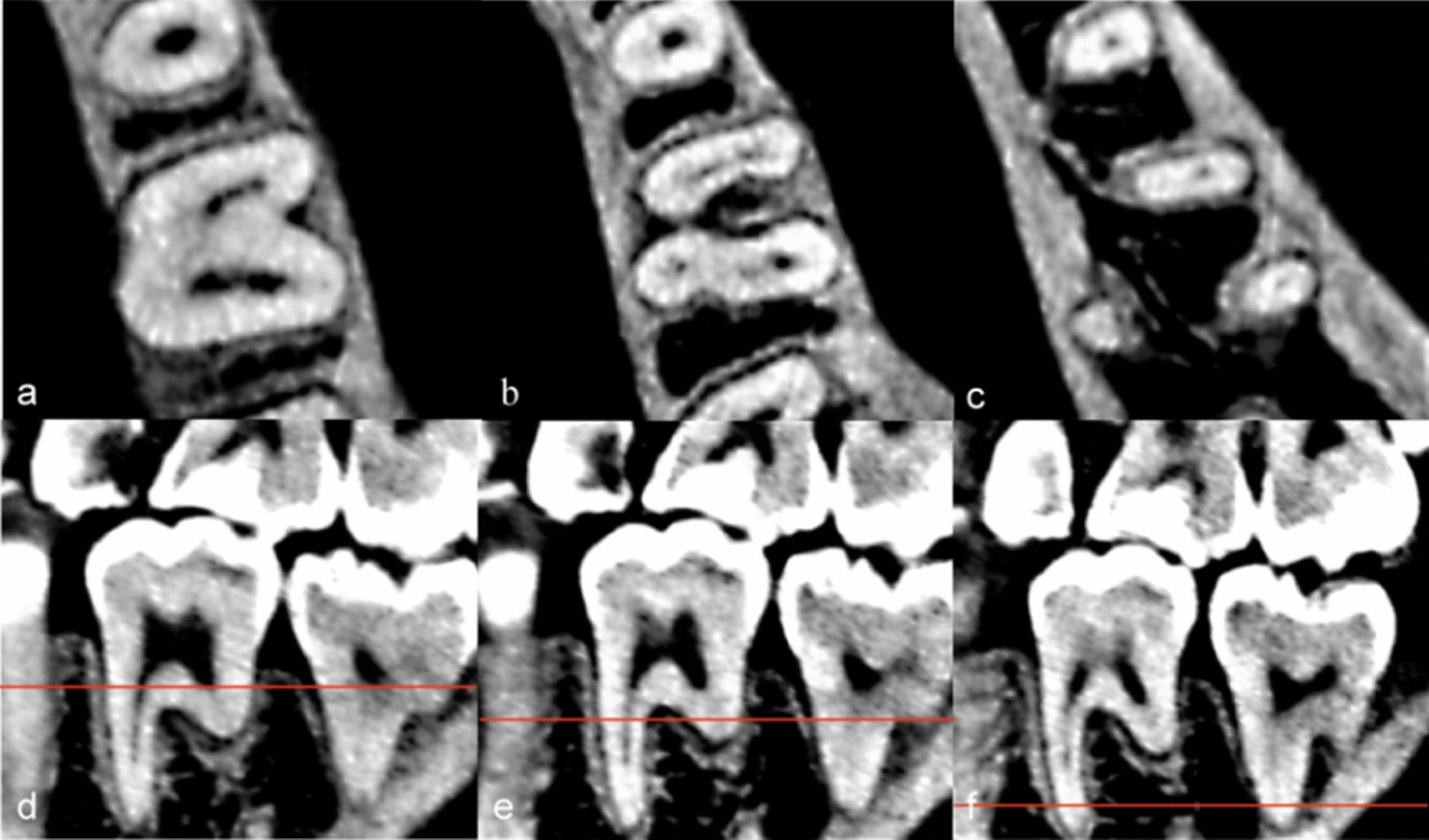
CBCT images of the 3-2-1 root canal system type. **a**–**c** were CT images with different cross sections: **a** Three canals can be seen at upper 1/3 of root canal; **b** There were two canals at middle1/3 of root canal; **c** two root canals eventually fused into one apical foramen; **d**–**f** are sagittal images

**Fig. 3:**
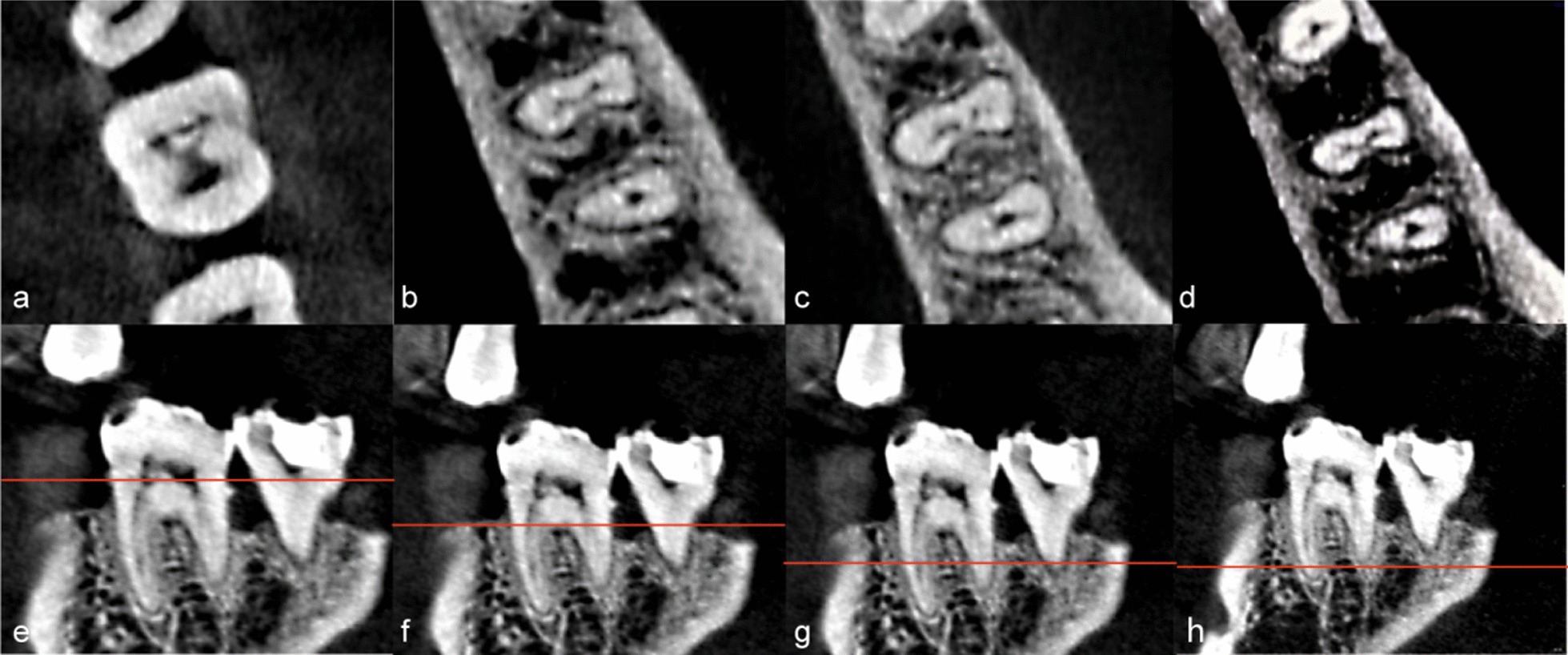
CBCT images of the 3-2-3-2 root canal system type. **a**–**d** were CT images with different cross sections: **a** There were three canals at pulp chamber floor; **b** The root canal can be seen from three canals to two canals; **c** Two canals branched into three canals; **d** Root canals eventually became two different apical foramens; **e**–**h** are sagittal images

**Fig. 4:**
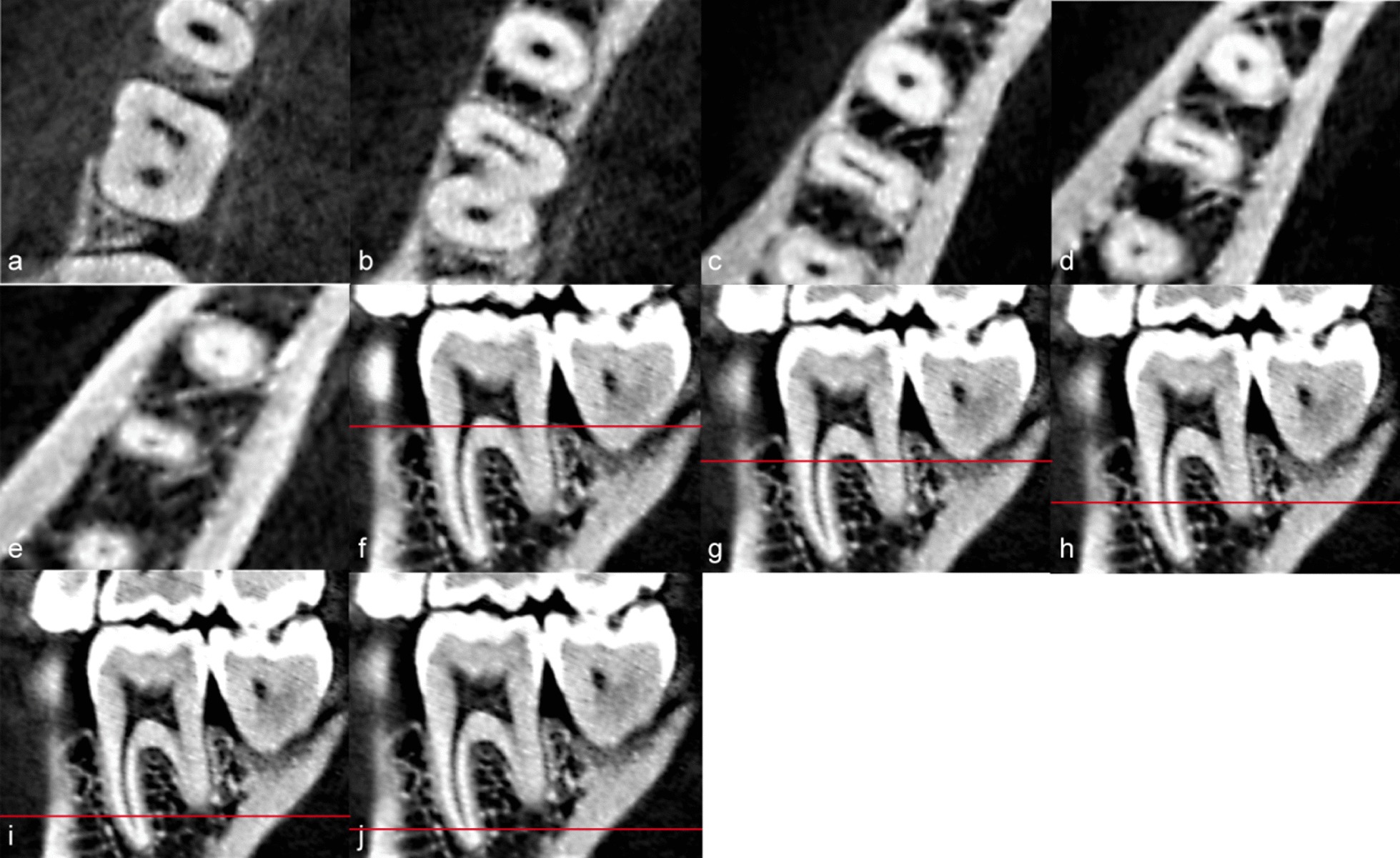
CBCT images of the 3-4-3-2-1 root canal system type. **a**–**e** were CT images with different cross sections: This type is more complicated, there are three root canals at the bottom of the pulp chamber, then four branches are branched, and fused into three root canals, then two canals can be seen at apical 1/3 of root canal. The root canals eventually fused into one apical foramen; **f**–**j** sagittal images

**Fig. 5:**
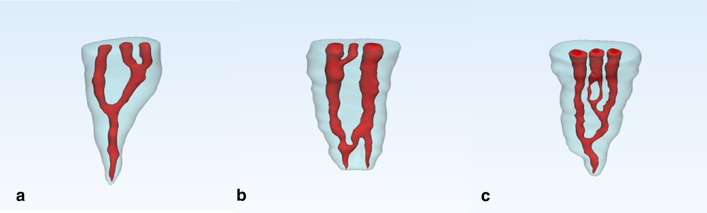
3D reconstruction images. **a** 3D reconstruction images of Type VIII (3-2-1); **b** 3D reconstruction images of Type IX (3-2-3-2); **c** 3D reconstruction images of Type X (3-4-3-2-1)

In this study, we classified the types of mesial root canals of 158 MFMs containing the MMCs and named them based on the Vertucci classification. However, these classifications are only suitable for the southern Chinese subpopulation. A total of ten types of root canals system were found. The most common type was type II (3-2) (53.80%) followed by type IV (2-3-2) (25.32%) and type I (type VIII) (8.23%). The other seven types were relatively rare. We found that three of these root canal types have not been reported in the previous literature using CBCT to study MFMs. Therefore, we showed them through CBCT screenshots and 3D reconstruction images (Table 4; Figs. 2, 3, 4, 5). As shown in Table 4, most of the MMCs were fused in the middle or apical part of the root canal for mesiobuccal root canals or mesiolingual root canals (92.41%). Only 8.23% of the MMCs were independent from the bottom of the pulp chamber to the apical foramen.

**Table 4:** Frequency distribution of the improved Vertucci’s classifications of middle mesial canal of mandibular first molars in a Southern Chinese subpopulation

Classification	Improved Vertucci’s classification	Number of specimens	Incidence (%)
Type I	Type VIII	12	8.23
Type II	3-2	85	53.80
Type III	2-3-1	4	2.53
Type IV	2-3-2	40	25.32
Type V	2-3-2-1	5	3.16
Type VI	1-2-3-2	2	1.27
Type VII	1-3-4-1	3	1.90
Type VIII	3-2-1*	3	1.90
Type IX	3-2-3-2*	2	1.27
Type X	3-4-3-2-1*	1	0.63

*We found that there were three classifications have not been reported by using CBCT

Among the root canal types found in this experiment, type I (type VIII), type II (type 3-2), type VIII (type 3-2-3-2), type IX (type 3-2-3-2) and type X (type 3-4-3-1) all have three root canal orifices at the bottom of the pulp chamber, namely, mesiobuccal (MB) root canal orifices, MM root canal orifices and mesiolingual (ML) root orifices. We measured the distance between the mesiobuccal and middle mesial orifices as well as the mesiolingual and middle mesial orifices in the above types in group A and group B, separately. The statistical results revealed no significant difference in the distance between the MMCs and the two main root canals in groups A and B (*P* > 0.05) (Table 5).

**Table 5:** The distance between the mesiobuccal—middle mesial orifices as well as the mesiolingual—middle mesial orifices

Classification	Improved Vertucci’s classification	MM-MB distance (Mean ± S)		MM-ML distance (Mean ± S)	
		< 40	≥ 40	< 40	≥ 40
Type I	Type VIII	1.55 ± 0.30	1.41 ± 0.25	1.56 ± 0.17	1.32 ± 0.20
Type II	3-2	1.44 ± 0.31	1.40 ± 0.28	1.41 ± 0.25	1.49 ± 0.28
Type VIII	3-2-1*	1.07 ± 0.27	1.01	1.17 ± 0.38	1.21
Type IX	3-2-3-2*	–	1.93 ± 0.04	–	1.49 ± 0.01
Type X	3-4-3-2-1*	1.02	–	1.02	–

*We found that there were three classifications have not been reported by using CBCT

As shown in Table 6, 95.15% and 71.84% of the MMCs in group A showed a significant curvature of > 10° in the mesiodistal and buccolingual directions, respectively. In group B, 96.36% of the MMCs showed a significant curvature of > 10° in the mesiodistal directions, and 85.45% in the buccolingual directions. By Chi-square test, the curvature in the mesiodistal directions was significantly greater than that in the buccolingual directions all in groups A and B, and the difference was statistically significant (*P* < 0.01). The curvature in the mesiodistal direction was 29.39 ± 8.53° in group A and 26.06 ± 8.50° in group B, and this difference was significant (*P* < 0.05). The curvature in the buccolingual direction was 21.34 ± 10.41° for group A and 22.45 ± 10.67° for group B, and this difference was not significant (*P* > 0.05) (Table 6).

**Table 6:** Curvature in the mesiodistal and buccolingual direction of the MMCs of mandibular first molars

Age	Direction	Slight (≤ 10°)	Medium (10°–30°)	Severely (> 30°)	Mean ± S
< 40	Mesiodistal	5 (4.85%)	62 (60.19%)	36 (34.95%)	29.39 ± 8.53
n = 103	Buccolingual	29 (28.16%)	54 (52.43%)	20 (19.42%)	21.34 ± 10.41
≥ 40	Mesiodistal	2 (3.64%)	37 (67.27%)	16 (29.09%)	26.06 ± 8.50
n = 55	Buccolingual	8 (14.55%)	30 (54.55%)	17 (30.91%)	22.45 ± 10.67

The incidences of mesiodistal and buccolingual curved regions of the MMCs were significantly higher in the middle 1/3 of the root canal than in the upper 1/3 and apical 1/3 of the root canal in groups A and B (Table 7).

**Table 7:** The curved regions of mesiodistal and buccolingual directions of the MMCs of the mandibular first molars

Age	Direction	Upper 1/3 (*P* ≤ 0.5)	Middle 1/3 (0.5 < *P* ≤ 2)	Apical 1/3 (*P* > 2)
< 40	Mesiodistal	9 (8.74%)	80 (77.67%)	14 (13.59%)
n = 103	Buccolingual	5 (4.85%)	92 (89.32%)	6 (5.83%)
≥ 40	Mesiodistal	2 (3.64%)	53 (96.36%)	0 (0%)
n = 55	Buccolingual	1 (1.82%)	51 (92.73%)	3 (5.45%)

## Discussion

The MMC, which is typically described as the presence of an additional canal in the mesial root, is an anatomic variant in MFMs. In accordance with previous studies, it showed that there have been noted for considerable difference in the MMC frequency. The detection rate of MMCs in mandibular first molars ranged from 0.26 to 46.15% [11–16], and the trait is noted in specific races. CBCT images are analysed in this experiment. 1100 patients, total in 1750 CBCT images at we found the incidence of MMC in mandibular first molars was 9.03%, and other reported results are also within this range. It can also be seen from our images that although it is a medium field of view image, MMCs can still be seen. The incidence rate of MMC may be higher if CBCT images with small field of vision and higher pixel size are used. No significant differences in the prevalence of MMCs in MFMs were noted between men and women. The results indicate that the incidence of MMCs in the mesial root of mandibular first molars decreases with age. Teeth experience age-related changes, such as tooth wear, periodontal degeneration, secondary dentin, root canal calcification, thickening of cementum, root resorption and increased transparent dentin in root, caused by external stimuli, such as caries and trauma. In this study, the high prevalence of MMCs among the subpopulation older than 40 years old was consistent with previous studies. Navid [27] and Fogel [28] showed that the incidence of coronal calcification of the root canal was increased in individuals older than 40 years old compared with those younger than 40 years old. This finding was also the reason why 40 years of age was the cut-off point for the two groups. The detection rate of MMCs decreased significantly in individuals older than 40 years old. This decreased detection rate was not attributed to a decrease in the actual existence rate of MMCs but was attributed to calcification of the root canal and secondary dentin hyperplasia, which reduced the diameter of the MMC root canal [28]. The reduction in space increases the difficulty of probing the MMC, which will also increase the difficulty of performing a CBCT scan in this experiment [29].

The mesial root of mandibular first molars exhibits significant variations, and various studies have investigated the morphology and anatomic complexity of the mesial root of mandibular first molars [7, 11, 30]. However, only a few studies used CBCT images to examine the prevalence and configuration of MMCs. Some studies report that middle mesial canals are located equidistant to both the main canals, whereas other studies report that these canals are closer to one of the main canals. The results of this study also revealed no marked difference in the distance between the mesiobuccal-middle mesial orifices and the mesiolingual-middle mesial orifices based on the above classifications, and a previous study reported similar results [31]. This measurement might help clinicians locate the MMC position. Locating MMCs is based on midpoint distance between MB and ML root canal orifice.

The mean curvature of MMCs in different age groups ranged from moderate to severe, and a root canal with large curvature posed significant difficulties in clinical operations. However, the root canal curvature of the mesiodistal direction decreased as age increased, which may be related to the increase in the calcification degree of root canals as age increases. The above studies demonstrate the correlation between MMC and age. Therefore, clinicians can design different treatment schemes based on patient age before treatment. In our study, a high proportion of patients of all ages exhibited curvature in mesiodistal and buccolingual directions as described by Perlea [32]. This finding suggested that the curvature of the root canal is typically three-dimensional. Clinicians should pay attention to the possibility of teeth curvature in the mesiodistal and buccolingual directions, not only one direction.

Table 7 demonstrates that the curved region of MMCs mostly occurs in the middle 1/3 of the canal, so this part should receive attention in the treatment process. However, the curved region of the root canal is also located at the upper 1/3 and apical 1/3 of this root canal. These regions are curved and narrow, so endodontic instruments easily break in these regions during root canal therapy [33]. Therefore, clinicians can improve the success rate of root canal therapy by knowing the shape and curvature of the root canal in advance. Given that few scholars at home and abroad study the curvature and curved region of MMCs, limited information is available, demonstrating the necessity and importance of our results.

The presence of MMCs should be considered before treatment. We identified 10 mesial root classification categories in mandibular first molars with middle mesial root canals. In most current articles, MMCs were classified according to Pomeranz et al. [9]; however, we classified these 10 root canal types based on the improved Vertucci classification. This categorization had several merits. This category contained information about root number and the distribution of root canals. This root canal classification can accurately and clearly present the trend of root canals. Our results revealed that the majority of MMCs were type II (3-2), which was consistent with previously reported studies of western Chinese [34], Korean [11], and Brazilian [28] populations. An increased prevalence of type IV (2-3-2) MMCs was found in our study, which is also similar to findings from western Chinese [34] and Korean [11] populations, but the incidence of this classification is lower in Brazilian and Turkish populations. These differences in root canal anatomy may be attributed to the influence of ethnic background on mandibular first molar root morphology. In the present investigation, type VIII, type II (3-2), and type IV (2-3-2) were the most common classifications. Clinicians should take these classifications into consideration during root canal treatments. A total of 27 variants were found in a study [35] that used microCT, and these difference may be caused by different study methods. These variants revealed complex variation in MMCs, which may pose challenges in surgical and non-surgical treatments. The presence of 3 canals that merge before the apical foramen and join to form 1 or 2 apical foramens has a minor effect on the treatment outcome. Ali’s research showed MMC orifices were at the CEJ level between the MB and ML canal orifices; thus, they would not require troughing to be located. MMCs would require troughing for 1–2 mm in depth to be accessed. However, the MMC orifices were deeper than 2 mm and could not be accessed by troughing preparation [36]. The chance of failure increases due to the remaining organic tissue and microorganisms close to the apical foramen [37]. The detection of an extra mesial canal is also important for the success of nonsurgical and surgical root canal treatments. Thus, information about the root canal configuration of MMC is important. The experimental methods for evaluating root canal morphology include clearing and staining, sectioning, conventional radiographs, magnification, micro–computed tomographic imaging, and mixed methods. In this study, we found 3 new root canal classifications in a southern Chinese subpopulation using CBCT. CBCT reconstruction software was used to slowly move the cross-section of each layer to clearly visualize the root canal branch and fusion. Thus, the advantages of CBCT in non-invasive observation of root canal system are completely realized. We cannot exclude the existence of additional new root canal classifications. We should study these classifications in more depth. Nevertheless, clinicians can use this type of complex root canal classification with the help of CBCT to adopt a more suitable treatment plan and prevent root canal treatment failure.

## Conclusion

The present CBCT study assessed the root canal configurations of mandibular first molars in a Chinese population, revealing the incidence of MMCs. Moreover, the results present new information on how to locate an MMC. Clinicians must consider the possibility of such anatomic variations.

## Data Availability

The datasets used and/or analysed during the current study are available from the corresponding author on reasonable request.

## Abbreviations

CBCT: Cone-beam computed tomography
MMC: Middle mesial canal
MFMs: Mandibular first molars
DLR: Distolingual root
MB: Mesiobuccal
ML: Mesiolingual

## References

[1] Vertucci FJ (1984). Root canal anatomy of the human permanent teeth. Oral Surg Oral Med Oral Pathol.

[2] Song M, Kim HC, Lee W, Kim E (2011). Analysis of the cause of failure in nonsurgical endodontic treatment by microscopic inspection during endodontic microsurgery. J Endod.

[3] Vertucci FJ (2005). Root canal morphology and its relationship to endodontic procedures. Endod Top.

[4] Yousuf W, Khan M, Mehdi H (2015). Endodontic procedural errors: frequency, type of error, and the most frequently treated tooth. Int J Dent.

[5] Shemesh A, Levin A, Katzenell V, Ben Itzhak J, Levinson O, Avraham Z (2017). C-shaped canals—prevalence and root canal configuration by cone beam computed tomography evaluation in first and second mandibular molars—a cross-sectional study. Clin Oral Investig.

[6] Zhang X, Xu N, Wang H, Yu Q (2017). A cone-beam computed tomographic study of apical surgery–related morphological characteristics of the distolingual root in 3-rooted mandibular first molars in a Chinese population. J Endod.

[7] De Pablo ÓV, Estevez R, Péix Sánchez M, Heilborn C, Cohenca N (2010). Root anatomy and canal configuration of the permanent mandibular first molar: a systematic review. J Endod.

[8] Barker BCW, Parsons KC, Mills PR, Williams GL (1974). Anatomy of root canals. III. Permanent mandibular molars. Aust Dent J.

[9] Pomeranz HH, Eidelman DL, Goldberg MG (1981). Treatment considerations of the middle mesial canal of mandibular first and second molars. J Endod.

[10] Karapinar-Kazandag M, Basrani BR, Friedman S (2010). The operating microscope enhances detection and negotiation of accessory mesial canals in mandibular molars. J Endod.

[11] Kim SY, Kim BS, Woo J, Kim Y (2013). Morphology of mandibular first molars analyzed by cone-beam computed tomography in a Korean population: variations in the number of roots and canals. J Endod.

[12] Azim AA, Deutsch AS, Solomon CS (2015). Prevalence of middle mesial canals in mandibular molars after guided troughing under high magnification: an in vivo investigation. J Endod.

[13] Banode AM, Gade V, Patil S, Gade J (2016). Endodontic management of mandibular first molar with seven canals using cone-beam computed tomography. Contemp Clin Dent.

[14] Sharma P, Shekhar R, Sharma A (2016). Endodontic management of mandibular first molar with six canals using CBCT-report of a case. J Clin Diagn Res JCDR.

[15] Martins JNR, Anderson C (2015). Endodontic treatment of the mandibular first molar with six roots canals: two case reports and literature review. J Clin Diagn Res.

[16] Jabali AH (2018). Middle mesial and middle distal canals in mandibular first molar. J Contemp Dent Pr.

[17] Scarfe WC, Levin MD, Gane D, Farman AG (2009). Use of cone beam computed tomography in endodontics. Int J Dent.

[18] Kumar V, Gossett L, Blattner A, Iwasaki LR, Williams K, Nickel JC (2011). Comparison between cone-beam computed tomography and intraoral digital radiography for assessment of tooth root lesions. Am J Orthod Dentofac Orthop.

[19] Patel S, Dawood A, Whaites E, Pitt Ford T (2009). New dimensions in endodontic imaging: part 1. Conventional and alternative radiographic systems. Int Endod J.

[20] Shukla S, Chug A, Afrashtehfar KI (2017). Role of cone beam computed tomography in diagnosis and treatment planning in dentistry: an update. J Int Soc Prev Commun Dent.

[21] Mirmohammadi H, Mahdi L, Partovi P, Khademi A, Shemesh H, Hassan B (2015). Accuracy of cone-beam computed tomography in the detection of a second mesiobuccal root canal in endodontically treated teeth: an ex vivo study. J Endod.

[22] Ng YL, Aung TH, Alavi A, Gulabivala K (2001). Root and canal morphology of Burmese maxillary molars. Int Endod J.

[23] Gulabivala K, Opasanon A, Ng YL, Alavi A (2002). Root and canal morphology of Thai mandibular molars. Int Endod J.

[24] Sert S, Bayirli GS (2004). Evaluation of the root canal configurations of the mandibular and maxillary permanent teeth by gender in the Turkish population. J Endod.

[25] Sert S, Şahinkesen G, Topçu FT, Eroǧlu ŞE, Oktay EA (2011). Root canal configurations of third molar teeth. A comparison with first and second molars in the Turkish population. Aust Endod J.

[26] Schneider SW (1971). A comparison of canal preparations in straight and curved root canals. Oral Surg Oral Med Oral Pathol.

[27] Akbarzadeh N, Aminoshariae A, Khalighinejad N, Palomo JM, Syed A, Kulild JC (2017). The association between the anatomic landmarks of the pulp chamber floor and the prevalence of middle mesial canals in mandibular first molars: an in vivo analysis. J Endod.

[28] Liu N, Li X, Liu N, Ye L, An J, Nie X (2013). A micro-computed tomography study of the root canal morphology of the mandibular first premolar in a population from southwestern China. Clin Oral Investig.

[29] Jaju PP, Jaju SP (2014). Clinical utility of dental cone-beam computedtomography: current perspectives. Clin Cosmet Investig Dent.

[30] Celikten B, Tufenkci P, Aksoy U, Kalender A, Kermeoglu F, Dabaj P (2016). Cone beam CT evaluation of mandibular molar root canal morphology in a Turkish Cypriot population. Clin Oral Investig.

[31] Weinberg EM, Pereda AE, Khurana S, Lotlikar PP, Falcon C, Hirschberg C (2020). Incidence of middle mesial canals based on distance between mesial canal orifices in mandibular molars: a clinical and cone-beam computed tomographic analysis. J Endod.

[32] Perlea P, Nistor CC, Imre M, Gheorghiu IM, Iliescu AA (2017). Middle mesial canal of the permanent mandibular first molars: an anatomical challenge directly related to the outcome of endodontic treatment. Rom J Morphol Embryol.

[33] Fuentes R, Farfán C, Astete N, Navarro P, Arias A (2018). Distal root curvatures in mandibular molars: analysis using digital panoramic X-rays. Folia Morphol.

[34] Wang Y, Zheng QH, Zhou XD, Tang L, Wang Q, Zheng GN (2010). Evaluation of the root and canal morphology of mandibular first permanent molars in a western chinese population by cone-beam computed tomography. J Endod.

[35] Versiani MA, Ordinola-Zapata R, Keleş A, Alcin H, Bramante CM, Pécora JD (2016). Middle mesial canals in mandibular first molars: a micro-CT study in different populations. Arch Oral Biol.

[36] Keleş A, Keskin C (2017). Detectability of middle mesial root canal orifices by troughing technique in mandibular molars: a micro-computed tomographic study. J Endod.

[37] Ratanajirasut R, Panichuttra A, Panmekiate S (2018). A cone-beam computed tomographic study of root and canal morphology of maxillary first and second permanent molars in a Thai population. J Endod.

